# The effect of tracheotomy on drug consumption in patients with acute aneurysmal subarachnoid hemorrhage: an observational study

**DOI:** 10.1186/s12871-015-0029-5

**Published:** 2015-04-08

**Authors:** Leiv Arne Rosseland, Jon Narum, Audun Stubhaug, Ulf Kongsgaard, Wilhelm Sorteberg, Angelika Sorteberg

**Affiliations:** 1 Department of Anesthesiology, Division of Emergencies and Critical Care, Oslo University Hospital – Rikshospitalet, Oslo, 0027 Norway; 2 Institute of Clinical Medicine, Faculty of Medicine, University of Oslo, Oslo, Norway; 3 Department of Neurosurgery, Oslo University Hospital – Rikshospitalet, Oslo, Norway

**Keywords:** Tracheotomy, Subarachnoid hemorrhage, Blood pressure, Stroke, Critical care, Midazolam, Fentanyl, Noradrenaline, Dopamine

## Abstract

**Background:**

Patients with aneurysmal subarachnoid hemorrhage (aSAH) are common in intensive care units (ICU). In patients with aSAH, sedation is used as a neuroprotective measure in order to secure adequate cerebral perfusion pressure (CPP). Compared with the use of an endotracheal tube, a tracheotomy has the advantage of securing the airway at a much lower level of distress, and aSAH patients can often be awakened more rapidly. Little is known about the impact of tracheotomy on the consumption of sedative/analgesic and vasoactive drugs and the maintenance of CPP within defined limits in aSAH patients.

**Methods:**

We conducted an observational study of aSAH patients who underwent percutaneous tracheotomy. A prospective registry of patient data was supplemented with retrospective retrievals from medical records. Sedative, analgesic and vasoactive drug doses were registered for 3 days prior to and after percutaneous tracheotomy, respectively. Blood pressure, CPP, and the mode of mechanical ventilation were registered 24 h prior to and after tracheotomy.

**Results:**

Between January 2001 and June 2009, 902 aSAH patients were admitted to our hospital; 74 (8%) were deeply comatose/dying upon arrival. The ruptured aneurysm was repaired in 828 patients (surgical repair 50%) and percutaneous tracheotomy was performed 182 times in 178 patients (59 men and 119 women). This subpopulation (178 of 828 patients) was significantly older (56 vs. 53 years) and presented with a more severe Hunt & Hess grade (p < 0.001). Percutaneous tracheotomy caused a marked decline in mean daily consumption of the analgesics/sedatives fentanyl, midazolam, and propofol, as well as the vasoactive drugs noradrenaline and dopamine. These declines were statistically and clinically significant. The mean CPP was 76 mmHg (SD 8.6) the day before and 79 mmHg (SD 9.6) 24 h after percutaneous tracheotomy. After percutaneous tracheotomy, mechanical ventilatory support could be reduced to a patient-controlled ventilatory support mode in a significant number of patients (p < 0.001).

**Conclusions:**

Percutaneous tracheotomy in aSAH patients is a swift procedure with low risk that is associated with a significant decline in the consumption of sedative/analgesic and vasoactive drugs while clinical surveillance parameters remain stable or improve.

## Background

Patients with aneurysmal subarachnoid hemorrhage (aSAH) are common in neurocritical care departments. In conjunction with sedation and continuous monitoring in the intensive care unit (ICU), early aneurysm repair and adequate management of acute hydrocephalus are crucial parts of initial treatment. Local management guidelines include sedation protocols, regulation of arterial blood pressure (ABP), and maintenance of intracranial pressure (ICP) below the defined critical threshold. Adequate cerebral perfusion pressure (CPP), which is defined by the difference between mean ABP and mean ICP, is a cornerstone of optimal management. Following aneurysm repair, our departmental guidelines consider CPP >70 mmHg to be the goal for most aSAH patients. In the presence of severe cerebral vasospasm, the CPP threshold can be raised to >90 mmHg.

Sedation with opioids, propofol, or benzodiazepines invariably lowers mean ABP, and the administration of vasoactive drugs may be required to maintain the target CPP. Depending on the severity of brain injury, the patient’s co-morbidities, and day-to-day assessment of the optimal treatment strategy, the patient may need secure airway access and ventilatory support. While most ICU patients need sedation and pain relief, sedation is also used as a neuroprotective measure in patients with aSAH. When sedated, the brain consumes less oxygen. Furthermore, deeper sedation represents one aspect of preventing or treating increases in ICP.

When the patient regains consciousness he or she may experience discomfort related to the endotracheal tube. When such a stress response is induced, ICP will increase. Frequent coughing may also increase ICP, and can cause unintended excessive cerebrospinal fluid (CSF) drainage through external CSF drains. These situations may necessitate increased sedation. Compared with the use of an endotracheal tube, a tracheotomy has the advantage of securing the airway at a much lower level of distress, and the aSAH patient can often be awakened more rapidly. Little is known about the impact of a tracheotomy on the consumption of sedative/analgesic and vasoactive drugs and the maintenance of CPP within the defined limits. In order to answer these questions, we performed the present observational study on aSAH patients that were managed using a percutaneous tracheotomy (PT).

## Methods

The Regional Committee for Medical and Health Research Ethics for South-East Norway approved the protocol of this study, and the study was exempted from the regulations of informed consent by the Norwegian Directorate of Health. The presentation of the study is prepared according to the STROBE guidelines [1].

All aSAH patients admitted to our hospital between January 2001 and June 2009 were acquired from a prospective registry. Some of these patients represent a sub-group of a previously published analysis in a general ICU population (N = 1000) [2]. The total patient population in our ICU is a mixture of medical and surgical intensive care cases, and the approximate mean number of annual ventilator days during this time period was 4,000. Aneurysmal SAH was the most frequent diagnosis, followed by heart failure, non-aneurysmal intracerebral hemorrhage, and intracerebral or intracerebellar infarction.

Anesthesiologists direct the ICU, and also performed all PTs. The decision of whether and when to establish a PT was based on clinical grounds by the neurosurgeon and the anesthesiologist in charge. Reduced sedation and wake-up were considered once the aneurysm was secured and the patient was stable and well within defined monitoring thresholds. Wake-up and extubation of the orotracheal tube as soon as feasible was one of the primary treatment goals. However, elevated ICP, serious cerebral vasospasm, and/or respiratory problems were indicative of a prolonged time interval before it would be possible to awaken the patient, and hence prompted consideration of tracheotomy. Severely impaired lung function with inspired oxygen fraction >60% or positive end expiratory pressure >10 cmH_2_O, led to postponement of PT until lung function improved, as would any cerebral instability, in particular increased ICP. The PT was performed by an experienced consultant or by a registrar supervised by a senior consultant in the ICU using a well established technique described elsewhere [3]. A third anesthesiologist secured the airway using direct laryngoscopy and fiber-optic bronchoscopy. The staff reported all PTs to the principal investigator, who consecutively recorded patient demographic data, primary diagnosis, the number of days on mechanical ventilation, duration of the procedure, and complications during the procedure and the following 48 h. Details regarding the aSAH patients were acquired from another registry and included demographic data, Hunt and Hess grade [4] prior to aneurysm repair or prior to sedation, angiographic classification, and mode of aneurysm repair. The sedative/analgesic and vasoactive drug doses were registered during the final 3 days prior to and the first 3 days after PT. Mean ABP, mean ICP, CPP, and the mode of mechanical ventilation were registered the day before and 24 h after the PT. Mode of mechanical ventilation was graded as 1 = spontaneous ventilatory support, 2 = partly controlled ventilation and patient-triggered ventilation, and 3 = controlled ventilation without patient triggering. The day of PT intervention was excluded from the analyses because the procedure requires variable amounts of analgesics and anesthetics. Each patient’s height and weight were measured, reported by relatives, or estimated by the intensive care doctor or nurse. The body mass index (BMI) was calculated based on these values. We used 90-day mortality rates for our analysis. The date of demise originating from the National Population Registry-Norway was collected from the patients’ electronic records.

### Statistical analysis

Demographic data, continuous numerical variables, and proportions are presented as mean and standard deviation or standard error, as median and range, or as interquartile range, where appropriate. Proportions were analyzed using the Chi-square test or Fisher’s exact test (specified in the results when relevant). The before- and after-effects of the mode of mechanical ventilation were tested statistically using the test for related marginal homogeneity in SPSS® version 21 for Windows (Statistical Packages for the Social Sciences), Chicago, IL. Sex differences were tested statistically with an independent sample *t*-test if normally distributed, or a Mann–Whitney *U* test. Risk factor analyses were performed using a linear regression model or Chi-square test. The statistical tests were performed with SPSS® version 18 for Windows, Chicago, IL. Figures were created with SigmaPlot® version 12.5 for Windows (SigmaPlot®^,^ Windows, Systat Software, San Jose, CA).

## Results

A total of 902 patients with aSAH were admitted to the hospital during the 8.5-year study period. Seventy-four (8%) of these patients did not have their aneurysm secured because they were deeply comatose/dying upon arrival. Among the 828 patients that underwent aneurysm repair, PT was performed 182 times in 178 patients (59 men and 119 women, Table 1). Three patients underwent PT twice, and one patient went through a third PT. The mean (SD) duration of mechanical ventilation before PT was 5.3 (2.8) days, and the mean (SD) interval from ictus to PT was 5.8 (4.2) days.

**Table 1:** **Aneurysmal subarachnoid hemorrhage patient characteristics**

		Non PT				PT			sign
**Number**		650				178			
**Female: Male** (% female)		431:219 (66.3)				119:59 (66.9)			NS
**Age** (range in years)		53 *(1 – 87)*				56 *(21 – 78)*			p = 0.005
**Surgical aneurysm repair** (%)		42.9				50.0			
**HH grade**		**1 - 3**	**4 -5**			**1 - 3**	**4 - 5**		
(%)		80.6	19.4			27.0	73.0		p < 0.001
**Location of ruptured aneurysm**	**MCA**	**ACA**	**ICA**	**BA**	**MCA**	**ACA**	**ICA**	**BA**	
(%)	21.7	34.9	22.2	6.8	21.3	37.1	8.5	6.2	NS

PT; percutaneous tracheotomy, HH; clinical grade before securing of the aneurysm/sedation according to Hunt and Hess [4] presented as good grade (HH grade 1-3) and poor grade (HH grade 4 and 5), MCA; Middle cerebral artery, ACA; Anterior Cerebral Aretery, including anterior communicating artery, ICA; Internal carotid artery, BA; basilar artery, sign; statistical significance.

The mean PT procedural time was 12.5 min (SD = 8.6, median 10 min, range 1–50 min). There was no procedure-related mortality. Difficulties during insertion of cannula, guide wire, or tracheal tube were registered in 19 patients, and minor bleeding was registered in 11 patients. One procedure was complicated by a short loss of airway control, one patient had perioperative hypoxia in spite of airway control, and one patient developed pneumothorax.

The distributions of sex, age, intervention, preoperative Hunt & Hess grade, and location of the ruptured aneurysm in patients with and without PT are shown in Table 1. The PT patients were older (mean age 56 years vs. 53 years, p = 0.005) and presented with more severe Hunt & Hess grade (p < 0.001). They were in poorer clinical condition prior to aneurysm repair; more than 70% presented with Hunt & Hess grade 4 or 5, compared with 19% of the non-PT patient population. Patient characteristics among men and women in the PT group are displayed in Table 2. Severity on admission was higher in the female population as 76% had Hunt & Hess grade 4 or 5 compared to 70% in men (Table 1). In the PT population, surgical aneurysm repair was performed in 50% of patients (Table 1). The 90-day mortality rate was 25% (95% CI 18.3 – 31.1).

**Table 2:** **Percutaneous tracheotomy patient characteristics**

	Male	Female	significance
Age (years)	*53 (33–77)*	*59 (21–78)*	NS
Height (cm)	181 (6.2)	167 (4.6)	NS
Weight (kg)	87 (15)*(60–128)*	69 (11)*(51–115)*	NS
BMI (kg/m^2^)	27 (4)	24 (3)	NS
Baseline HH grade	*4* (IQR 2)	*4* (IQR 1.25)	NS

Demographic data of patients presented as mean (standard deviation), *median and range,* or inter quartile range (IQR). BMI; body mass index, HH; Hunt Hess grade [4].

**Figure 1:**
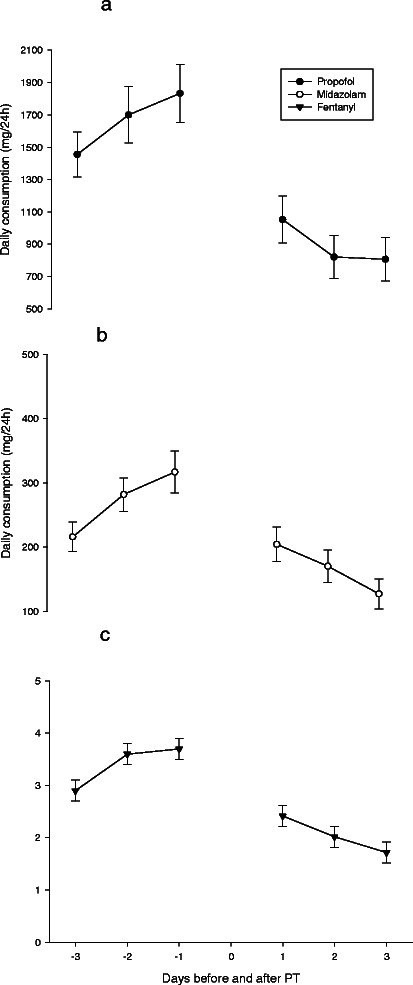
**Daily consumption of sedative/analgesic drugs.** Daily consumption of fentanyl **(a)**, midazolam **(b)**, and propofol **(c)** (mg/24 h) presented as mean (SEM) during the 3 days before and the 3 days after tracheotomy.

**Figure 2:**
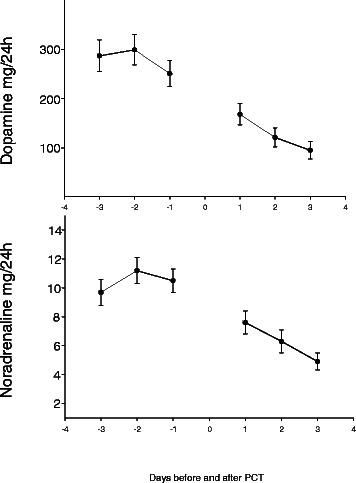
**Daily consumption of vasoactive drugs.** Daily consumption of dopamine and noradrenaline (mg/24 h) presented as mean (SEM) during the 3 days before and the 3 days after tracheotomy.

**Figure 3:**
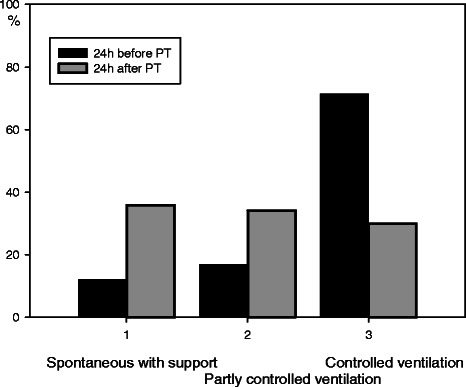
**Mechanical ventilator mode 24 h before and after tracheotomy.** The modes of mechanical ventilation were graded as follows: 1 = spontaneous ventilatory support mode; 2 = partly controlled ventilation and patient-triggered ventilation; and 3 = controlled ventilation without patient triggering. Data were registered 24 h before (black columns) and 24 h after (grey columns) tracheotomy.

Mean daily consumption of fentanyl, midazolam, and propofol tended to increase during the 3 days immediately before PT, but decreased significantly during the 3 days immediately after PT (Table 3, Figure 1). Likewise, mean daily consumption of noradrenaline and dopamine dropped significantly after PT and continued to decline during the 3 days after PT (Figure 2). Mean CPP was 76 mmHg (SD 8.6) the day before and 79 mmHg (SD 9.6) 24 h after PT (p < 0.001, paired sample *t*-test). Median mechanical ventilator mode was 3 (controlled ventilation) (interquartile range 2–3) before PT and 2 (spontaneous ventilation) (interquartile range 1–3) after PT (p < 0.001; Figure 3).

**Table 3:** **Administration of sedative drugs before and after PT**

	Before PT	After PT	sign
Fentanyl (mg/day)	3.5 (2.7)	2.4 (3.9)	p = 0.001
Midazolam (mg/day)	277 (315)	168 (289)	p < 0.001
Propofol (mg/day)	1618 (1687)	842 (1475)	p < 0.001

Fentanyl, midazolam, and propofol presented as mean (standard deviation) of the three last days before PT and the three days after PT.

## Discussion

The main findings of the present study were that percutaneous tracheotomy (PT) can be performed safely in aSAH patients and that consumption of analgesics, sedatives, and vasoactive drugs became markedly reduced after PT.

### Percutaneous tracheotomy

During the past decade, PT has become a routine method in the ICU. Randomized trials have documented a lower incidence of bleeding and infections compared with open surgical tracheotomy [5-8]. PT is cost-effective [9] and can be performed in a shorter procedure time than surgical tracheotomy [8], with minimal risk of increasing ICP [10]. Our present finding of a median procedural time of 10 min and few adverse events corroborates these reports. Regarding cost-effectiveness, the present study did not investigate outcome or total hospital time. Nevertheless, the drastic reduction in analgesics/sedatives and vasoactive drugs observed after PT may counterbalance some or all of the cost of performing PT.

Timing of the tracheotomy in ventilated patients has been the focus of some randomized trials [11]. One randomized controlled trial recently reported similar complication rates and patient outcomes in early versus late PT [12]. Because the benefits of timely tracheotomy in the patient population with aSAH may differ from those in the general ICU population, it would have been of interest to perform controlled comparisons in aSAH patients or to analyze this subgroup of patients in the large randomized controlled trials that have already been performed. However, because even the biggest randomized controlled trial addressing the timing of tracheotomy included only a small number of patients with intracranial pathology, a new, prospective, randomized study in this patient population will be necessary.

### Vasoactive drugs

Aneurysmal SAH is a life-threatening event, associated with a mortality rate as high as 75% in poor grade patients [13,14]. Some of this mortality can be attributed to delayed cerebral ischemia. Hence, optimizing the CPP (usually >70 mmHg) is a central part of the treatment protocol, and sedated patients most often require vasoactive drugs to obtain the prescribed CPP. The safety of vasoactive drugs regarding the cerebral vasculature is not well documented; therefore, titrating vasoactive drug doses as low as possible is mandatory. This is particularly important regarding the most feared complication of aSAH: cerebral vasospasm. One might raise concerns that the use of vasoactive drugs could exacerbate and/or prolong cerebral vasospasm. However, little is known about the effect of vasoactive drugs on the human intracranial vasculature. One randomized study conducted by Rondeau et al. has compared the combination of noradrenaline and dobutamine with noradrenaline alone [15]. They hypothesized that the dobutamine-induced increase in cardiac output might reduce the incidence of intracranial vasospasm, but failed to show any difference. Experimental data in rabbits indicate that noradrenaline-induced hypertension may increase the diameter and flow in vasospastic intracranial vessels [16]. Our clinical experience supports that noradrenaline is safe, although the benefit of high-dose noradrenaline is controversial.

Phenylephrine represents an alternative to noradrenaline and is commonly used to increase blood pressure to target CPP in neurointensive care [17]. Dopamine is less used [18], but may be indicated if monotherapy with noradrenaline causes bradycardia. Adrenaline should not be used, as it may cause vasospasm [19]. Regardless, the rate of possible vasoactive drug-induced adverse events will be lower if the target CPP can be maintained at lower levels of vasoactive drugs. The present study demonstrates that PT had a profound impact on vasoactive drug levels, with a marked and ongoing drop in drug usage during the first 3 days after PT.

Because cerebral vasospasm may lead to delayed cerebral ischemia and is a major contributor to poor outcome [20], prophylaxis and treatment of vasospasm are imperative. The neurointensivist will often face the dilemma between optimizing the CPP and minimizing the vasoactive and sedative/analgesic drug doses, especially if the targeted CPP must be increased further owing to documented severe vasospasm. In the present study, after PT, the CPP was easier to control; the mean ICP remained low even when the patient was less sedated. This was likely caused by the reduced level of stress associated with a PT versus an orotracheal tube.

### Sedative/analgesic drugs

A retrospective analysis of 312 patients in a general ICU population documented that administration of sedatives decreased after PT [21], while the observed decrease prior to and after tracheotomy in a 48-h perspective in a study of 1,780 patients did not reach statistical significance [22]. Our study solely comprises aSAH patients, and the fact that these patients require ICP control may have had an impact on our findings. Many aSAH patients experience severely reduced consciousness after the ictus, and prolonged mechanical ventilation is necessary. Sedation continues during invasive ventilatory support, as guided by surveillance thresholds for ICP, CPP, blood oxygenation, and other parameters. We observed a statistically and clinically significant decrease in the consumption of sedative/analgesic drugs after PT. The doses of fentanyl and midazolam were larger than in a general ICU population [21], and the decrease in drug doses was greater than expected and could not be attributed to the expected natural course of disease. In contrast, the use of sedatives/analgesics was increasing during the 3 days prior to PT. At our ICU, the amount of sedatives used in aSAH patients is usually reduced as soon as is feasible; however, if such a reduction leads to ICP and/or CPP beyond the desired thresholds, sedation is again increased. Hence, tracheotomized patients experience less discomfort when their level of consciousness is increasing, compared with patients having an orotracheal tube. Both discomfort and arousal would contribute to elevations in ICP that in turn would be counteracted with increased sedative/analgesic use. This mechanism, unique for neurointensive patients, in particular those with aSAH, might explain the increasing use of sedatives/analgesics during the days prior to PT, in contrast to a general ICU population [22].

Our study suggests that PT is particularly well indicated in the aSAH population because it allows ICP and ABP control with much smaller amounts of sedative/analgesic drugs and opioids. This makes it difficult to detect severe vasospasm, and clinical signs are easier to monitor if the patient is awake. Documented serious vasospasm was treated with deep sedation and elevation of the CPP threshold in excess of 90 mmHg. No PTs were performed during the phase of serious cerebral vasospasm or other neurological instabilities. After PT, we observed a concomitant increase in CPP that was probably caused by the rapid decrease in sedatives/analgesics, given that there were no changes in the prescribed target CPP values. Although the increase in CPP was statistically significant, it was probably not of clinical impact.

Reduced sedation was associated with changing the ventilator mode; however, a retrospective analysis does not allow conclusions regarding this synergy. The fact that a significant number of patients went from controlled mode to spontaneous ventilation after PT was likely due to the greatly reduced opioid doses.

### Population-related factors

The present 3-month mortality rate of 25% was below the average in our general ICU population (30%) [2]. Mortality was also low compared with previously published data [23,24], particularly considering our high fraction of patients who presented with Hunt & Hess grades 4 and 5 (“poor grade”). Mortality in this subgroup is reportedly as high as 75% [13,14]. The observation of a significantly poorer clinical grade in the patient population that underwent PT is expected, because patients with an anticipated long course of disease were more likely to be selected for PT. Aneurysmal SAH is more common in women [25,26]; correspondingly, more women underwent PT. The reason for a higher fraction of women being in a poorer clinical state prior to aneurysm repair is not obvious from the available data, but may be related to age, pre-existing co-morbidity, delays in seeking medical attention, larger hemorrhages, or possibly more problems with acute hydrocephalus.

### Limitations of the study

The available observational data cannot answer the question of whether tracheotomy improved outcome or had an effect on the duration of hospitalization in this patient population. Although retrospective analyses may document associations and generate hypotheses, they cannot prove causation. A randomized trial with multifactorial analysis is necessary to investigate these aspects of PT.

Regrettably, the ICU did not register sedation and agitation scores for the patients during the entire study period. This limits our ability to interpret our findings regarding drug consumption. Likewise, GCS could not be retrieved for all days before and after PT in all patients.

## Conclusions

Percutaneous tracheotomy in aSAH patients is a swift procedure with low risk that is associated with a significant decline in the consumption of sedative/analgesic and vasoactive drugs while clinical surveillance parameters remain stable or improve.

## Abbreviations

aSAH: Aneurysmal subarachnoid hemorrhage
ICU: Intensive care unit
CPP: Cerebral perfusion pressure
ABP: Arterial blood pressure
ICP: Intracranial pressure
CSF: Cerebrospinal fluid
PT: Percutaneous tracheotomy
BMI: Body mass index
